# *N*-Acetyl-L-cysteine in human rheumatoid arthritis and its effects on nitric oxide (NO) and malondialdehyde (MDA): analytical and clinical considerations

**DOI:** 10.1007/s00726-022-03185-x

**Published:** 2022-07-13

**Authors:** Dimitrios Tsikas, Marie Mikuteit

**Affiliations:** 1 Core Unit Proteomics, Institute of Toxicology, Hannover Medical School, Carl-Neuberg-Str. 1, 30625 Hannover, Germany; 2 Clinic for Rheumatology und Immunology, Hannover Medical School, Carl-Neuberg-Str. 1, 30625 Hannover, Germany

**Keywords:** Artefacts, Guidelines, Malondialdehyde, Nitric oxide, Nitrite, Nitrate, Oxidative stress, Peer reviewing, Rheumatoid arthritis, Sample storage

## Abstract

*N*-Acetyl-L-cysteine (NAC) is an endogenous cysteine metabolite. The drug is widely used in chronic obstructive pulmonary disease (COPD) and as antidote in acetaminophen (paracetamol) intoxication. Currently, the utility of NAC is investigated in rheumatoid arthritis (RA), which is generally considered associated with inflammation and oxidative stress. Besides clinical laboratory parameters, the effects of NAC are evaluated by measuring in plasma or serum nitrite, nitrate or their sum (NOx) as measures of nitric oxide (NO) synthesis. Malondialdehyde (MDA) and relatives such as 4-hydroxy-nonenal and 15(*S*)-8-*iso*-prostaglandin F_2α_ serve as measures of oxidative stress, notably lipid peroxidation. In this work, we review recent clinico-pharmacological studies on NAC in rheumatoid arthritis. We discuss analytical, pre-analytical and clinical issues and their potential impact on the studies outcome. Major issues include analytical inaccuracy due to interfering endogenous substances and artefactual formation of MDA and relatives during storage in long-term studies. Differences in the placebo and NAC groups at baseline with respect to these biomarkers are also a serious concern. Modern applied sciences are based on data generated using commercially available instrumental physico-chemical and immunological technologies and assays. The publication process of scientific work rarely undergoes rigorous peer review of the analytical approaches used in the study in terms of accuracy/trueness. There is pressing need of considering previously reported reference concentration ranges and intervals as well as specific critical issues such as artefactual formation of particular biomarkers during sample storage. The latter especially applies to surrogate biomarkers of oxidative stress, notably MDA and relatives. Reported data on NO, MDA and clinical parameters, including C-reactive protein, interleukins and tumour necrosis factor α, are contradictory in the literature. Furthermore, reported studies do not allow any valid conclusion about utility of NAC in RA. Administration of NAC patients with rheumatoid arthritis is not recommended in current European and American guidelines.

## Introduction

*N*-Acetyl-L-cysteine is an endogenous metabolite of L-cysteine metabolism, i.e., *N*-acetylation mainly in the kidney. The plasma concentration of endogenous NAC in healthy humans is about 100 nM (Liu et al. 2010). NAC is one of the most widely used drugs worldwide with many areas of pharmacological applications. Adverse effects are mostly gastro-intestinal symptoms but appear rarely. The pharmacological effects of NAC are primarily based on the reactivity of its sulfhydryl (SH) group (p*K*_a_^COOH^, 3.24; p*K*_a_^SH^, 9.5) against various functionalities (Borgström et al. 1986; Holdiness 1991; Tenório et al. 2021). NAC is mainly used as a mucolytic drug in chronic respiratory diseases such as chronic obstructive pulmonary disease (COPD) to decrease viscosity by reducing disulphide (S–S) groups in proteins (Calverley et al. 2021). NAC is also used as an antidote of acetaminophen (paracetamol) intoxication to supply L-cysteine (Cys) by enzymatic *N*-deacetylation. Cys is an essential precursor required in the biosynthesis of glutathione (GSH), which in turn is required in the GSH *S*-transferase detoxification of the toxic acetaminophen intermediate *N*-acetyl-*p*-benzoquinone imine (NAPQI).

As a mucolytic, NAC is orally administered at standard doses of 600 mg per day and reaches circulating maximum concentrations of about 15 µM after 40 min and urinary excretion rates of about 27 µmol/12 h (Borgström et al. 1986). Oral administration of a single 600 mg NAC effervescent tablet to five healthy young volunteers (10 mg/kg) increased the mean plasma NAC concentration from about 0.17 µM (limit of quantitation) to 4.6 µM after 60 min, which then decreased to 2.5 µM after 30 min (Tsikas et al. 1998). The corresponding concentrations of Cys were 10.0 µM, 18.6 µM, 17.4 µM, indicating Cys as a major NAC metabolite of pharmacological NAC (Tsikas et al. 1998). For intravenous application, such as in paracetamol poisoning, considerably higher amounts of NAC are used because of the need to detoxify large amounts of toxic acetaminophen metabolites, notably NAPQI. Intravenous administration of 600 mg (3.7 mmol) NAC resulted in plasma concentrations of about 300 µM with a mean elimination half-life of 2.3 h (Borgström et al. 1986). Even at high oral doses (≥ 600 mg/day), NAC is considered pharmacologically safe (Calverley et al. 2021; Kobroob et al. 2021). The oral bioavailability of NAC is very low (6–10%) in humans (Borgström et al. 1986; Holdiness 1991; Tenório et al. 2021). Chemical esterification of NAC to its ethyl ester (NACET), a highly lipophilic and freely permeable substance, improves its pharmacokinetic and pharmacodynamic properties, including oral bioavailability in the rat, reactivity towards disulphide groups and supply of Cys and H_2_S (Giustarini et al. 2012; Tsikas et al. 2018a).

## Oral NAC in rheumatic diseases in humans

The role of NAC in rheumatic diseases has been only sporadically explored. There are few publications on clinical NAC use in rheumatoid arthritis (RA). In an open trial on seven patients with refractory RA, no beneficial effects were observed after up to 12 months of treatment with oral NAC (600–1200 mg/d) (Jonsson et al. 1986). In RA, the utility of oral NAC (2 × 2000 mg/day for 1 week) was tested in gold toxicity (Vreugdenhil and Swaak 1990). NAC was proposed to have useful therapeutic potential in RA patients with limited interstitial lung disease (Kelly and Saravanan 2008). More recently, NAC is increasingly investigated as a pharmacological strategy in the activation of the metabolic control of the immune system in rheumatic diseases in humans (Perl 2017). NAC has been used in a clinico-pharmacological study to treat patients with systemic lupus erythematosus (SLE) (Lai et al. 2012), a chronic inflammatory disease. Patients had lower GSH concentrations in peripheral blood lymphocytes (PBL) than healthy controls. Upon NAC administration, GSH concentrations increased in PBL, but not in blood. Administration of high doses of NAC (2.4 g/day and 4.8 g/day, but not 1.2 g/day) improved disease activity, presumably by regulating mammalian target of rapamycin (mTOR) by NAC-derived GSH (Lai et al. 2012; Wyman and Perl 2020). NAC also stimulates protein synthesis in enterocytes independently of glutathione synthesis (Yi et al. 2016). An indication was observed that oral administration of NAC (2 × 600 mg/d for 12 weeks) to RA patients improved their subjective health status (Batooei et al. 2018). The authors suggested NAC as an adjuvant therapy. Yet, in a nationwide cohort study in Taiwan, patients who took oral NAC were found to have increased risk of knee osteoarthritis (Yeh et al. 2020). In patients with RA, NAC administration (2 × 600 mg/d for 8 weeks) was found to exert beneficial effects in clinical characteristics of RA (Jamali et al. 2021). In the current European (Smolen et al. 2020) and American (Fraenkel et al. 2021) guidelines on the treatment of RA, the additional treatment with NAC has not been recommended.

In recent clinico-pharmacological studies on NAC (Zhang et al. 2018; Hashemi et al. 2019; Esalatmanesh et al. 2022), clinical laboratory measurements were accompanied with measurements of biochemical parameters to study potential underlying mechanisms. Measured parameters included nitrite and nitrate as indicators of nitric oxide (NO) synthesis and malondialdehyde (MDA) as an indicator of oxidative stress (lipid peroxidation). These studies are discussed in more detail with respect to NO and MDA in the next section.

In this article, we will not focus on clinical chemistry laboratory parameters related to inflammation and oxidative stress such as C-reactive protein (CRP), IL-6 and TNF-α. Yet, we should mention that reported results in human studies are contradictory. Zhang and colleagues reported decrease in TNF-α in the NAC group (Zhang et al. 2018). Hashemi and colleagues reported no differences between NAC and placebo groups with respect to CRP, IL-6 and TNF-α (Hashemi et al. 2019). Esalatmanesh and colleagues reported no differences between the NAC and placebo groups with respect to hs-CRP (Esalatmanesh et al. 2022).

## Measurement of NO and MDA in human rheumatic diseases

NO and oxidative stress are assumed to be involved in rheumatic diseases (Jang and Murrell 1998). In this context and in addition to specific biochemical indicators of inflammation, the NO metabolites nitrite and nitrate may be under certain conditions useful measures of systemic whole-body NO synthesis (Sütö et al. 1995; Baylis and Vallance 1998; Tsikas 2005, 2015). Circulating and urinary MDA usually serve as a measure of systemic and whole-body oxidative stress, notably lipid peroxidation (Tsikas 2017).

Nitrite, nitrate, and the sum of nitrite and nitrate (NOx) are often measured by commercially available spectrophotometric ELISA kits, which are based on the Griess reaction after enzymatic reduction of nitrate (Tsikas 2007). MDA and other longer aldehydes and lipid peroxides are commonly measured by spectrophotometric assays as the thiobarbituric acid reactive substances (TBARS) (Tsikas 2017). Spectrophotometric Griess- and TBARS-based are subject of numerous interferences and artefacts, which may compromise severely the analytical quality and the study outcome (Tsikas 2007, 2017). Potential artefacts in TBARS measurement in studies using exogenous antioxidants have also been reported (Lin and Jamieson 1994). Interferences may be of particular importance in placebo-control studies, as placebo formulations do not contain NAC that could potentially interfere. Such analytical concerns and concentrations reported in previous studies for healthy subjects, i.e., references values and intervals, have either not been adequately considered or even ignored.

## Administration of NAC in clinical studies measuring NO metabolites and MDA

In a randomized controlled trial, patients with community-acquired pneumonia were additionally treated with NAC (Zhang et al. 2018). In the study, the NAC and non-NAC groups were found to have very close baseline MDA plasma concentrations of (3.20 ± 1.43 µM versus 3.14 ± 1.66 µM, *P* = 0.689). Seven days after oral intake of NAC (2 × 600 mg/day) by the patients of the NAC group resulted in plasma MDA concentrations of 2.01 ± 0.74 µM. The respective MDA plasma concentration in the non-NAC group after seven days was 2.71 ± 1.17 µM. The close comparability of the baseline MDA plasma concentrations in the NAC and non-NAC groups argues for a reduction of the oxidative stress/lipid peroxidation upon NAC treatment of the patients with community-acquired pneumonia (Zhang et al. 2018). The MDA plasma concentration decreased in both groups of the study after seven days storage of the plasma samples at − 80 °C until analysis. This may suggest that the age difference of the plasma samples of seven days may have influenced the extent of artefactual formation of MDA due to sample storage. Artefactual MDA formation is expected to be higher in the baseline samples compared to the plasma samples obtained after seven days when baseline and treatment samples are analyzed together at the end of studies (see below). This study has not reported results on NO synthesis (Zhang et al. 2018).

In a pilot placebo-controlled study, oral administration of NAC (2 × 600 mg/day for 12 weeks) in addition to the basic therapy to Iranian RA patients resulted in lower serum NO and MDA concentrations in the NAC group (*n* = 23) compared to the placebo group (*n* = 19) (Hashemi et al. 2019). In the study, serum NO was measured as nitrite + nitrate (i.e., the sum of nitrite and nitrate) using a commercially available ELISA assay, whereas serum MDA was measured using a spectrophotometric TBARS-based assay. This assay was performed as reported by another group for lipid peroxides in animal tissues yet not in serum or plasma (Ohkawa et al. 1979). NOx concentrations were lower in both groups after 12 weeks. Based on serum MDA measurements the authors concluded that oral NAC can significantly reduce (by 16%) several oxidative stress parameters in RA (Hashemi et al. 2019). Yet, the reported units for concentrations of serum NOx (about 5 mmol/ml) and serum MDA (about 500 mmol/ml) question the outcome of the study (Hashemi et al. 2019). The correct MDA and NOx serum concentrations would be rather of the order of 5 µM each.

In a randomized, double-blind, placebo-controlled trial, Iranian RA patients were treated with NAC in addition to the basic therapy (Esalatmanesh et al. 2022). Patients received for 12 weeks either NAC (2 × 600 mg/day, *n* = 34) or placebo (*n* = 36) twice a day. Several parameters including MDA and NOx were measured in serum samples at baseline and after 12 weeks.

The serum MDA concentration in the placebo group of the study was reported to be (mean ± SD) 2.21 ± 1.11 µM at baseline and 2.21 ± 0.98 µM after 12 weeks, indicating no change over time in the placebo group (*P* = 0.615) (Esalatmanesh et al. 2022). The serum MDA concentration in the NAC group was reported to be (mean ± SD) 4.24 ± 1.78 µM at baseline, but only 1.49 ± 1.59 µM after 12 weeks, indicating a strong decrease (65%) of oxidative stress upon NAC treatment (*P* < 0.001). These serum MDA concentrations are within the wide-ranges reported in the literature for healthy and ill subjects using various methodologies, including assays based on TBAR and gas chromatography–mass spectrometry (GC–MS) (Giustarini et al. 2009; Tsikas 2017). The comparison of the baseline MDA concentrations did not reveal statistical significance between the placebo and NAC groups, although the baseline MDA values were almost two times higher in the NAC than in the placebo group (Esalatmanesh et al. 2022).

The serum NO_x_ concentration in the placebo group of the study (Esalatmanesh et al. 2022) was reported to be [mean ± standard deviation (SD)] 2.77 ± 1.66 µM at baseline and 2.78 ± 1.79 µM after 12 weeks, indicating no change of the placebo treatment (*P* = 0.303). The serum NO_x_ concentration in the NAC group was reported to be (mean ± SD) 5.28 ± 2.36 µM at baseline and 1.14 ± 1.47 µM after 12 weeks, indicating a strong decrease (about 80%) of systemic NO synthesis upon NAC treatment (*P* < 0.001). The comparison of the baseline NO_x_ concentrations revealed statistical significance between the placebo and NAC groups, with the baseline NO_x_ values being almost two times higher in the NAC group (Esalatmanesh et al. 2022). Furthermore, the serum concentrations of NO_x_ reported for both groups of the study (Esalatmanesh et al. 2022) are very low when compared to the majority of reported values (Tsikas 2005, 2008) and in a general Iranian population (Ghasemi et al. 2008). Mean serum NOx concentrations of about 25 µM were reported in a general Iranian population of apparently healthy young and elderly subjects (age range 21–86 years) with very slight differences between men and women (Ghasemi et al. 2008). Specifically, serum NOx concentrations were (95% interval) 11.5–76.4 µM in men and 10.1–65.6 µM in women) as measured by a non-commercially available Griess assay, that includes reduction of nitrate to nitrite by vanadium (III) chloride (Ghasemi et al. 2010). Thus, reference serum NO_x_ concentrations in adult Iranian populations and in other populations are much higher (Tsikas 2005, 2007, 2008) than those reported for the Iranian patients (Hashemi et al. 2019; Esalatmanesh et al. 2022). These groups used the same commercially available colorimetric assay, which seems to lack satisfactory analytical reliability including assay range and sensitivity. Thus, after deproteinization and neutralization of serum samples, the serum NO_x_ concentrations measured in the NAC group after 12 weeks are of the order of 1.1 ± 1.5 µM and even below the sensitivity reported by the manufacturer (https://zellbio.eu/?s=nitrite). The manufacturer indicates that protein removal is required by ultrafiltration, yet this material is not included in the assay kit. Omitting this step is very likely to compromise the measurement of NOx in serum and plasma (Tsikas 2007).

In patients (*n* = 28) with rheumatic diseases including RA, we measured by GC–MS lower (by 28%) serum nitrate concentrations than in healthy controls (19.8 vs. 27.4 µM, *P* = 0.014), indicating lower systemic NO synthesis in RA (Pham et al. 2009). The urinary excretion of nitrite and nitrate did not differ between RA patients and healthy control groups, indicating comparable whole-body NO synthesis in RA patients and healthy controls (Kayacelebi et al. 2014). The serum NOx concentrations reported by Hashemi et al. 2019 and Esalatmanesh et al. 2022 using the same commercially available assay kits are much lower than the concentrations reported by many other groups in healthy and ill subjects (Tsikas 2005, 2007, 2008) including Iranian people (Ghasemi et al. 2008, 2010). In patients with chronic inflammatory rheumatic diseases including RA higher nitrite concentrations are found in synovial fluid as compared to serum (Farrell et al. 1992; Pham et al. 2009). This may indicate that nitrite in synovial fluid is better suitable than in serum or plasma in rheumatic diseases.

## Pre-analytical, non-analytical and study-design considerations of NO and MDA

Reportedly, there are remarkable discrepancies between studies with respect to reported serum/plasma nitrite, nitrate and NOx concentrations measured in the NAC and placebo groups at baseline and after treatment compared to those measured in healthy humans. This is especially the case when spectrophotometric assays based on the Griess reaction are used. Such assays often suffer from methodological problems, which have been discussed elsewhere in detail (Tsikas 2007, 2008, 2017).

An important, yet rarely considered pre-analytical issue in MDA analysis, especially in the context of long-term clinico-pharmacological studies, is that of the time regarding sample collection, storage and analysis. Non-consideration of this pre-analytical issue is likely to compromise severely the outcome of the study, even in placebo-controlled long-term clinico-pharmacological studies (Tsikas 2017).

The pre-analytical issue of sample storage may be a general concern when artefactual formation of analytes in biological samples occurs. This is especially the case for MDA in lipid-rich samples such as plasma, serum and tissue. Remarkable artefactual formation of MDA in plasma samples has been reported more than four decades ago (Lee 1980). The utility of plasma MDA as a measure of in vivo lipid peroxidation has been even questioned for this reason more than three decades ago (Hackett et al. 1988).

**Fig. 1:**
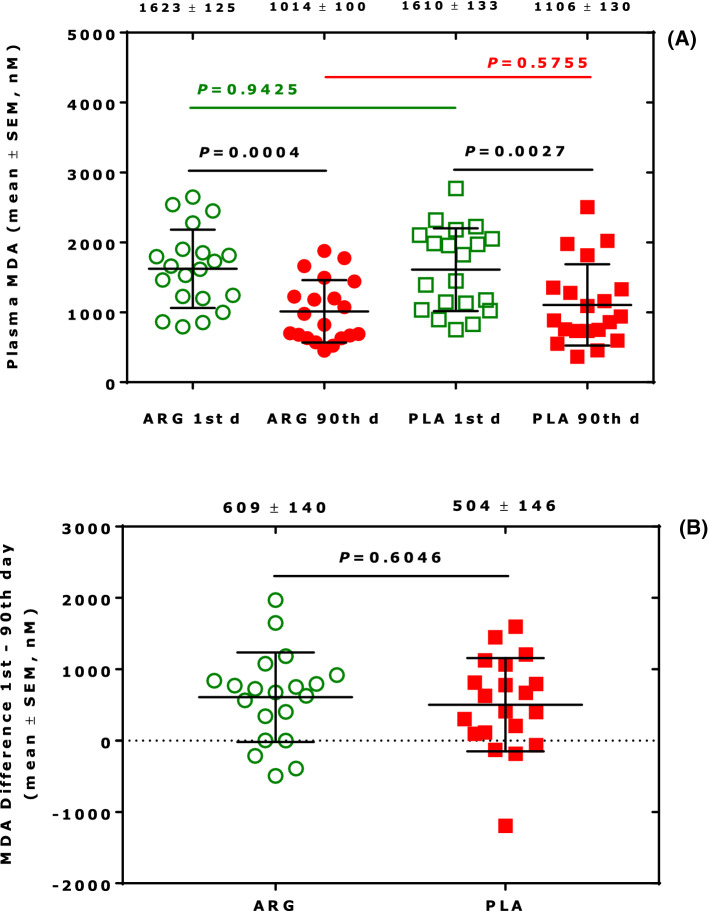
**A** Plasma MDA concentrations in the arginine (ARG) and placebo (PLA) groups at day 1 and day 90 upon oral administration of L-arginine or placebo to patients with peripheral arterial occlusive disease (PAOD). **B** Difference in the plasma MDA concentrations in the ARG and PLA groups at day 1 and day 90th upon oral administration of L-arginine or placebo to patients with PAOD (each *n* = 20). Statistical analysis was performed using unpaired *t* test. Numbers on the top are mean ± standard error of the mean of MDA concentrations and differences. The Figure was constructed with the data of Table 2 of a previously reported work (Tsikas 2017). *d* day

We have previously observed in a placebo, double-blind clinico-pharmacological study that plasma MDA concentrations were lower in the placebo and verum (drug) groups in patients with cardiovascular disease at the end of the study compared to baseline (Tsikas et al. 2016; Tsikas 2017). Results revealed that storage of plasma samples (at − 80 °C) leads to lower MDA concentrations in the plasma samples that were collected at the end of the study as compared to those collected at the beginning of the study (Fig. 1). Similar results were also obtained for 4-hydroxy-nonenal and the F_2_-isoprostane 15(*S*)-8-*iso*-prostaglandin F_2α_, two related biomarkers of oxidative stress (Tsikas 2017). There were no statistically significant differences in the groups with respect to MDA, 4-hydroxy-nonenal and 15(*S*)-8-*iso*-prostaglandin F_2α_ concentrations measured in concomitantly collected urine samples. Therefore, the higher plasma MDA concentrations measured at baseline are most likely due to artefactual formation of MDA in the plasma samples during the sample storage period, which was at least 12 weeks longer compared to those collected at the end of the study (Tsikas 2017).

**Fig. 2:**
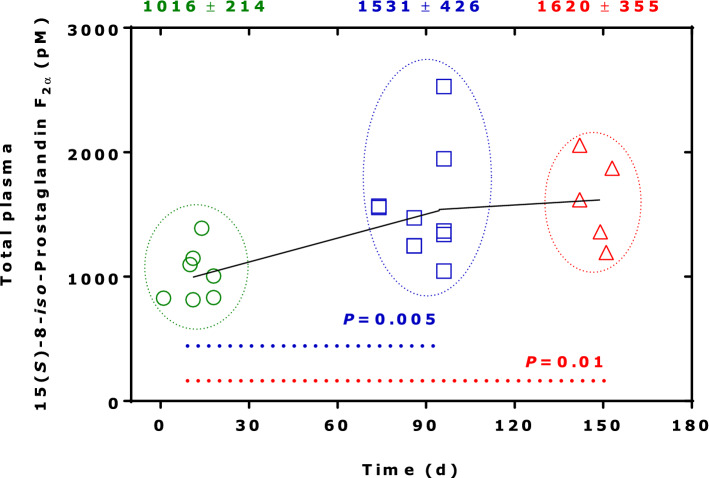
Time course of the concentration of total 15(*S*)-8-*iso*-prostaglandin F_2α_ (free acid and esterified to lipids) in aliquoted (1 ml) and at − 80 °C stored human plasma samples serving as the quality control (QC) sample over 153 days. 15(*S*)-8-*iso*-prostaglandin F_2α_ was determined by GC–MS/MS after immunoaffinity chromatography (IAC) column extraction and derivatization (Tsikas and Suchy 2016). *n* = 2 for day 1, day 10, day 18, and day 74; *n* = 3 for day 86; *n* = 5 for day 96; *n* = 1 each for days 149, 151 and 153, thus simulating a long-term clinical study. Unpaired *t* test was performed between three groups in the periods 1 to 18 days, 74–96 days, and 149–160 days, as indicated by the frames. Numbers on the top are mean ± standard error of the mean. *d* day

In the framework of clinico-pharmacological studies, we implemented quality control (QC) systems for endogenous analytes including NO metabolites (Tsikas 2008), MDA and 15(*S*)-8-*iso*-prostaglandin F_2α_ (Tsikas and Suchy 2016). We used such a system to investigate the artefactual formation of MDA and 15(*S*)-8-*iso*-prostaglandin F_2α_ in human plasma. An example is shown in Fig. 2 for total (i.e., free and esterified) 15(*S*)-8-*iso*-prostaglandin F_2α_ in pooled human plasma. It is obvious that 15(*S*)-8-*iso*-prostaglandin F_2α_ is artificially formed during storage in − 80 °C. It is also obvious that artefactual formation of 15(*S*)-8-*iso*-prostaglandin F_2α_ is very variable and seems to be a saturable process. 15(*S*)-8-*iso*-Prostaglandin F_2α_ is one of theoretically 64 prostaglandin F_2_ (PGF_2_) isomers. In human plasma, the concentration of PGF_2_ increased 50-fold after storage the plasma sample at -20 °C when compared to the fresh unfrozen plasma sample (Morrow et al. 1990).

Such QC systems are useful to determine the accuracy and precision by which endogenous substances are measured in biological samples at the time points of analysis (Tsikas 2008). Yet, they are not useful to correct for artefactual formation of analytes that possess the potential for artefactual formation during long storage periods, such as MDA and 15(*S*)-8-*iso*-prostaglandin F_2α_. Because of the potential of abundant artefactual formation, reporting detailed protocols including times and periods of sample collection, conditions of sample storage and final analysis ensures scientific visibility.

In long-term clinico-pharmacological studies, it is advisable first to determine baseline values of main biochemical parameters such as MDA, and to start the treatment only when there are no statistical differences between the groups with respect to MDA. Age- and gender-matching in placebo and verum groups may not suffice for successful clinico-pharmacological studies. An alternative could be the measurement of MDA in urine (Guichardant et al. 1994; Tsikas 2017). Yet, in contrast to 15(*S*)-8-*iso*-prostaglandin F_2α_ urinary MDA is rarely measured in clinical trials.

It is worth mentioning that human platelets are a major source of MDA, which can be inhibited by acetylsalicylic acid (aspirin) in parallel to thromboxane. In vitro, both cyclooxygenase I (COX-I) and cyclooxygenase II (COX-II) are producers of MDA, indicating that MDA is an enzymatic metabolite of arachidonic acid, which can be modulated by thiols including GSH, NAC and cysteine (Tsikas et al. 2012; Tsikas 2017). Platelets are associated with RA. MDA production is considered an index of platelet arachidonic acid metabolism. MDA concentrations measured in plasma of RA were found to be in normal range (Colli et al. 1982). Extra-cellular thiols such as Cys, NAC and GSH can modulate platelet function including platelet aggregation (Tsikas 2021). A prerequisite for pharmacological NAC to exert effects on platelets in vitro is its bioconversion GSH (Gibson et al. 2009), yet at concentrations that cannot be reached by regular oral doses of NAC.

Another important issue when measuring nitrite and nitrate in urine is acute drug-induced changes (Sütö et al. 1995). Single oral administration of NAC (600 mg) to healthy subjects resulted in temporary elevation of the urinary excretion of nitrate and nitrite (Tsikas et al. 2014). This observation suggests that NAC administration may inhibit renal carbonic anhydrase dependent reabsorption of nitrate and nitrite, thus simulating enhanced NO synthesis (Tsikas et al. 2018b).

In addition to the issues discussed above, it is notable that many expectations of effects of antioxidants and vitamins in clinico-pharmacological studies are rarely fulfilled (Giustarini et al. 2009). A reason for this could be that expectations are largely based on results observed in in vitro experiments mostly using supra-pharmacological/physiological concentrations that cannot be reached in vivo upon oral administration (Giustarini et al. 2009). This is likely to be the case in NAC, because the very low bioavailability of orally taken NAC does not allow reach mM-concentrations that are often used in experimental studies (e.g., Anfossi et al. 2001; Kanai et al. 2020). Even intravenous injection of 600 mg (3676 µmol) to healthy subjects does not result in plasma NAC concentrations higher than 300 µM, which decline with a half-life of about 2 h (Borgström et al. 1986).

## Conclusions and perspectives

A general phenomenon in the scientific literature are the remarkable differences in serum, plasma and urine concentrations of endogenous biochemical parameters (biomarkers) which are used as measures of health state, disease activity and effects of drugs. In patients with RA treated with NAC, investigated biomarkers are nitrite, nitrate and their sum (NO_x_) as measures of NO synthesis and MDA as a measure of oxidative stress, specifically lipid peroxidation. Large differences between studies make reliable comparison difficult, may question the outcome of studies, the efficacy of the pharmacological treatment, and the elucidation of mechanisms that may underlie the drug’s action. An important factor in long-term studies is the different age of lipid-rich samples, such as plasma and serum, at the time of analysis. Longer stored samples, for example those collected at baseline, may be an abundant source of severe analytical error for biomarkers such as MDA, 4-hydroxy-nonenal, 15(*S*)-8-*iso*-prostaglandin F_2α_ and relatives. This is because they are readily produced artefactually in considerable, hard to control extent during sample storage at frozen state, i.e., presumably independent of the drug’s action. Processing QC samples is a useful monitoring, but it is not suitable in quantitative analyses for correction of measurements for artefactual formation. Other strategies and protocols may be more efficient, such as more sophisticated study design and time for sample analysis.

As a matter-of-fact, measured concentrations of endogenous analytes are considered reliable independent of the laboratory, its analytical features and expertise. Nitrite, nitrate and MDA belong to those analytes, but the spectrum of analytes is certainly very wide. This practice is often facilitated by the commercial availability of “ready-to-use” assays. Not least importantly, the unavoidable biological variability is by nature a strong multiplier. Analytical measures obtained by uncritical use of commercially available assays are freely included in statistical analyses, and are used to draw conclusions and propose potential underlying mechanisms. Yet, considerable discrepancies between researcher groups with respect to particular biomarkers such as nitrite, nitrate and MDA generate uncertainty and doubts. Without question, NAC and its major metabolites Cys and GSH are antioxidants as they share the same SH functionality. Yet, the effects of orally administered NAC and its metabolites must not necessarily significantly add to increases in endogenous intra-cellular (e.g., GSH) and extra-cellular (e.g., Cys) stores. Taken these issues together, the final result of this practice is a kind of unwritten yet risky agreement that all published data are eventually reliable.

The adequacy of NAC in the treatment of RA when evaluated using surrogate biomarkers such as nitrite, nitrate and MDA warrants further clinico-pharmacological studies. Few available reports do not permit dependable evaluation, to a major part because of the use of doubtful analytical approaches. Often, the standard pharmacological treatment of patients with RA who participate in the study is not adequately reported, neither is a clinical outcome (such as DAS28) mentioned. Possible interactions of adverse drug effects of the standard RA therapy with the metabolism of NAC, MDA and NO should be considered. Also, the clinical effects of NAC should be measured, to compare the patients’ disease activity to the results of the blood analysis. Studies need to be well-designed and performed using carefully tested, reliable analytical methods for the measurement of nitrate, nitrite and MDA. Scientists need to consider previous knowledge of pre-analytical, analytical and non-analytical problems. Scientists need also to consider references values in particular populations. The publication process of scientific work generally needs to include peer review of the analytical approaches used in the studies. Measurement of MDA and other biomarkers of oxidative stress in urine is possible and may be a serious alternative to plasma and serum, because urine is poor in lipids and the potential of artefactual formation is negligible. Should large trials reveal that NO synthesis is lower in human RA than in healthy subjects and would oral NAC administration decrease NO synthesis, pharmacological NAC at high doses would be possibly contra-indicated in rheumatic diseases such as osteoarthritis. Current European and American guidelines do not include administration of NAC in addition to basic pharmacotherapy to patients with RA.

## Abbreviations

COPD: Chronic obstructive pulmonary disease
COX: Cyclooxygenase
GC–MS: Gas chromatography–mass spectrometry
GC–MS/MS: Gas chromatography–mass spectrometry–mass spectrometry
GSH: Glutathione
IAC: Immunoaffinity chromatography
MDA: Malondialdehyde
mTOR: Mammalian target of rapamycin
NAC: *N*-Acetyl-L-cysteine
NACET: *N*-Acetyl-L-cysteine ethyl ester
NAPQI: *N*-Acetyl-*p*-benzoquinone imine
NO: Nitric oxide
NOx: Nitrite plus nitrate
PBL: Peripheral blood lymphocytes
QC: Quality control
RA: Rheumatoid arthritis
SH: Sulfhydryl
SLE: Systemic lupus erythematosus
TBARS: Thiobarbituric acid reactive substances

## References

[CR1] Anfossi G, Russo I, Massucco P, Mattiello L, Cavalot F, Trovati M (2001). N-acetyl-L-cysteine exerts direct anti-aggregating effect on human platelets. Eur J Clin Invest.

[CR2] Batooei M, Tahamoli-Roudsari A, Basiri Z, Yasrebifar F, Shahdoust M, Eshraghi A, Mehrpooya M, Ataei S (2018). Evaluating the effect of oral N-acetylcysteine as an adjuvant treatment on clinical outcomes of patients with rheumatoid arthritis: a randomized, double blind clinical trial. Rev Recent Clin Trials.

[CR3] Baylis C, Vallance P (1998). Curr Opin Nephrol Hypertens.

[CR4] Borgström L, Kagedal B, Paulsen O (1986). Pharmacokinetics of N-acetylcysteine in man. Eur J Clin Pharmacol.

[CR5] Calverley P, Rogliani P, Papi A (2021). Safety of N-acetylcysteine at high doses in chronic respiratory diseases: a review. Drug Saf.

[CR6] Colli S, Maderna P, Tremoli E, Colombo F, Canesi B (1982). Platelet function in rheumatoid arthritis. Scand J Rheumatol.

[CR7] Esalatmanesh K, Jamali A, Esalatmanesh R (2022). Effects of *N*-acetylcysteine supplementation on disease activity, oxidative stress, and inflammatory and metabolic parameters in rheumatoid arthritis patients: a randomized double-blind placebo-controlled trial. Amino Acids.

[CR8] Farrell AJ, Blake DR, Palmer RM, Moncada S (1992). Increased concentrations of nitrite in synovial fluid and serum samples suggest increased nitric oxide synthesis in rheumatic diseases. Ann Rheum Dis.

[CR9] Fraenkel L, Bathon JM, England BR, St Clair EW, Arayssi T, Carandang K, Deane KD, Genovese M, Huston KK, Kerr G, Kremer J, Nakamura MC, Russell LA, Singh JA, Smith BJ, Sparks JA, Venkatachalam S, Weinblatt ME, Al-Gibbawi M, Baker JF, Barbour KE, Barton JL, Cappelli L, Chamseddine F, George M, Johnson SR, Kahale L, Karam BS, Khamis AM, Navarro-Millán I, Mirza R, Schwab P, Singh N, Turgunbaev M, Turner AS, Yaacoub S, Akl EA (2021). 2021 American college of rheumatology guideline for the treatment of rheumatoid arthritis. Arthritis Rheumatol.

[CR10] Ghasemi A, Zahedi Asl S, Mehrabi Y, Saadat N, Azizi F (2008). Serum nitric oxide metabolite levels in a general healthy population: relation to sex and age. Life Sci.

[CR11] Ghasemi A, Zahediasl S, Azizi F (2010). Reference values for serum nitric oxide metabolites in an adult population. Clin Biochem.

[CR12] Gibson KR, Neilson IL, Barrett F, Winterburn TJ, Sharma S, MacRury SM, Megson IL (2009). Evaluation of the antioxidant properties of N-acetylcysteine in human platelets: prerequisite for bioconversion to glutathione for antioxidant and antiplatelet activity. J Cardiovasc Pharmacol.

[CR13] Giustarini D, Dalle-Donne I, Tsikas D, Rossi R (2009). Oxidative stress and human diseases: origin, link, measurement, mechanisms, and biomarkers. Crit Rev Clin Lab Sci.

[CR14] Giustarini D, Milzani A, Dalle-Donne I, Tsikas D, Rossi R (2012). N-Acetylcysteine ethyl ester (NACET): a novel lipophilic cell-permeable cysteine derivative with an unusual pharmacokinetic feature and remarkable antioxidant potential. Biochem Pharmacol.

[CR15] Guichardant M, Valette-Talbi L, Cavadini C, Crozier G, Berger M (1994). Malondialdehyde measurement in urine. J Chromatogr B Biomed Appl.

[CR16] Hackett C, Linley-Adams L, Walker V (1988). Plasma malondialdehyde: a poor measure of in vivo lipid peroxidation. Clin Chem.

[CR17] Hashemi G, Mirjalili M, Basiri Z, Tahamoli-Roudsari A, Kheiripour N, Shahdoust M, Ranjbar A, Mehrpooya M, Ataei S (2019). A pilot study to evaluate the effects of oral N-acetyl cysteine on inflammatory and oxidative stress biomarkers in rheumatoid arthritis. Curr Rheumatol Rev.

[CR18] Holdiness MR (1991). Clinical pharmacokinetics of N-acetylcysteine. Clin Pharmacokinet.

[CR19] Jamali F, Ahmadzadeh A, Sahraei Z, Salamzadeh J (2021). Study of the effects of N-acetylcysteine on inflammatory biomarkers and disease activity score in patients with rheumatoid arthritis. Iran J Allergy Asthma Immunol.

[CR20] Jang D, Murrell GA (1998). Nitric oxide in arthritis. Free Radic Biol Med.

[CR21] Jonsson H, Wollheim FA, Svensson B (1986). No effect of acetylcystein on refractory rheumatoid arthritis. Clin Exp Rheumatol.

[CR22] Kanai T, Kondo N, Okada M, Sano H, Okumura G, Kijima Y, Ogose A, Kawashima H, Endo N (2020). The JNK pathway represents a novel target in the treatment of rheumatoid arthritis through the suppression of MMP-3. J Orthop Surg Res.

[CR23] Kayacelebi AA, Pham VV, Willers J, Hahn A, Stichtenoth DO, Jordan J, Tsikas D (2014). Plasma homoarginine (hArg) and asymmetric dimethylarginine (ADMA) in patients with rheumatoid arthritis: is homoarginine a cardiovascular corrective in rheumatoid arthritis, an anti-ADMA?. Int J Cardiol.

[CR24] Kelly C, Saravanan V (2008). Treatment strategies for a rheumatoid arthritis patient with interstitial lung disease. Expert Opin Pharmacother.

[CR25] Kobroob A, Peerapanyasut W, Kumfu S, Chattipakorn N, Wongmekiat O (2021). Effectiveness of N-acetylcysteine in the treatment of renal deterioration caused by long-term exposure to bisphenol A. Biomolecules.

[CR26] Lai ZW, Hanczko R, Bonilla E, Caza TN, Clair B, Bartos A, Miklossy G, Jimah J, Doherty E, Tily H, Francis L, Garcia R, Dawood M, Yu J, Ramos I, Coman I, Faraone SV, Phillips PE, Perl A (2012). N-acetylcysteine reduces disease activity by blocking mammalian target of rapamycin in T cells from systemic lupus erythematosus patients: a randomized, double-blind, placebo-controlled trial. Arthritis Rheum.

[CR27] Lee DM (1980). Malondialdehyde formation in stored plasma. Biochem Biophys Res Commun.

[CR28] Lin Y, Jamieson D (1994). Potential artifacts in TBARS measurement in tissues from animals treated with antioxidants. Biomed Environ Sci.

[CR29] Liu YM, Liu Y, Lu C, Jia JY, Liu GY, Weng LP, Wang JY, Li GX, Wang W, Li SJ, Yu C (2010). Relative bioavailability of generic and branded acetylcysteine effervescent tablets: a single-dose, open-label, randomized-sequence, two-period crossover study in fasting healthy Chinese male volunteers. Clin Ther.

[CR30] Morrow JD, Harris TM, Roberts LJ (1990). Noncyclooxygenase oxidative formation of a series of novel prostaglandins: analytical ramifications for measurement of eicosanoids. Anal Biochem.

[CR31] Ohkawa H, Ohishi N, Yagi K (1979). Assay for lipid peroxides in animal tissues by thiobarbituric acid reaction. Anal Biochem.

[CR32] Perl A (2017). Metabolic control of immune system activation in rheumatic diseases. Arthritis Rheumatol.

[CR33] Pham VV, Stichtenoth DO, Tsikas D (2009). Nitrite correlates with 3-nitrotyrosine but not with the F(2)-isoprostane 15(S)-8-iso-PGF(2alpha) in urine of rheumatic patients. Nitric Oxide.

[CR34] Smolen JS, Landewé RBM, Bijlsma JWJ, Burmester GR, Dougados M, Kerschbaumer A, McInnes IB, Sepriano A, van Vollenhoven RF, de Wit M, Aletaha D, Aringer M, Askling J, Balsa A, Boers M, den Broeder AA, Buch MH, Buttgereit F, Caporali R, Cardiel MH, De Cock D, Codreanu C, Cutolo M, Edwards CJ, van Eijk-Hustings Y, Emery P, Finckh A, Gossec L, Gottenberg JE, Hetland ML, Huizinga TWJ, Koloumas M, Li Z, Mariette X, Müller-Ladner U, Mysler EF, da Silva JAP, Poór G, Pope JE, Rubbert-Roth A, Ruyssen-Witrand A, Saag KG, Strangfeld A, Takeuchi T, Voshaar M, Westhovens R, van der Heijde D (2020). EULAR recommendations for the management of rheumatoid arthritis with synthetic and biological disease-modifying antirheumatic drugs: 2019 update. Ann Rheum Dis.

[CR35] Sütö T, Losonczy G, Qiu C, Hill C, Samsell L, Ruby J, Charon N, Venuto R, Baylis C (1995). Acute changes in urinary excretion of nitrite + nitrate do not necessarily predict renal vascular NO production. Kidney Int.

[CR36] Tenório MCDS, Graciliano NG, Moura FA, Oliveira ACM, Goulart MOF (2021). N-acetylcysteine (NAC): impacts on human health. Antioxidants (Basel).

[CR37] Tsikas D (2005). Methods of quantitative analysis of the nitric oxide metabolites nitrite and nitrate in human biological fluids. Free Radic Res.

[CR38] Tsikas D (2007). Analysis of nitrite and nitrate in biological fluids by assays based on the Griess reaction: appraisal of the Griess reaction in the L-arginine/nitric oxide area of research. J Chromatogr B Analyt Technol Biomed Life Sci.

[CR39] Tsikas D (2008). A critical review and discussion of analytical methods in the L-arginine/nitric oxide area of basic and clinical research. Anal Biochem.

[CR40] Tsikas D (2015). Circulating and excretory nitrite and nitrate: their value as measures of nitric oxide synthesis, bioavailability and activity is inherently limited. Nitric Oxide.

[CR41] Tsikas D (2017). Assessment of lipid peroxidation by measuring malondialdehyde (MDA) and relatives in biological samples: analytical and biological challenges. Anal Biochem.

[CR42] Tsikas D (2021). Extra-platelet low-molecular-mass thiols mediate the inhibitory action of S-nitrosoalbumin on human platelet aggregation via S-transnitrosylation of the platelet surface. Amino Acids.

[CR43] Tsikas D, Suchy MT (2016). Protocols for the measurement of the F2-isoprostane, 15(S)-8-iso-prostaglandin F2alpha, in biological samples by GC-MS or GC-MS/MS coupled with immunoaffinity column chromatography. J Chromatogr B Analyt Technol Biomed Life Sci.

[CR44] Tsikas D, Sandmann J, Ikic M, Fauler J, Stichtenoth DO, Frölich JC (1998). Analysis of cysteine and *N*-acetylcysteine in human plasma by high-performance liquid chromatography at the basal state and after oral administration of *N*-acetylcysteine. J Chromatogr B Biomed Sci Appl.

[CR45] Tsikas D, Suchy MT, Niemann J, Tossios P, Schneider Y, Rothmann S, Gutzki FM, Frölich JC, Stichtenoth DO (2012). Glutathione promotes prostaglandin H synthase (cyclooxygenase)-dependent formation of malondialdehyde and 15(S)-8-iso-prostaglandin F2α. FEBS Lett.

[CR46] Tsikas D, Niemann J, Flentje M, Schwarz A, Tossios P (2014). N-Acetylcysteine (NAC) inhibits renal nitrite and nitrate reabsorption in healthy subjects and in patients undergoing cardiac surgery: risk of nitric oxide (NO) bioavailability loss by NAC?. Int J Cardiol.

[CR47] Tsikas D, Rothmann S, Schneider JY, Suchy MT, Trettin A, Modun D, Stuke N, Maassen N, Frölich JC (2016). Development, validation and biomedical applications of stable-isotope dilution GC-MS and GC-MS/MS techniques for circulating malondialdehyde (MDA) after pentafluorobenzyl bromide derivatization: MDA as a biomarker of oxidative stress and its relation to 15(S)-8-iso-prostaglandin F2α and nitric oxide (NO). J Chromatogr B Analyt Technol Biomed Life Sci.

[CR48] Tsikas D, Schwedhelm KS, Surdacki A, Giustarini D, Rossi R, Kukoc-Modun L, Kedia G, Ückert S (2018). S-Nitroso-N-acetyl-L-cysteine ethyl ester (SNACET) and N-acetyl-L-cysteine ethyl ester (NACET)-Cysteine-based drug candidates with unique pharmacological profiles for oral use as NO, H_2_S and GSH suppliers and as antioxidants: results and overview. J Pharm Anal.

[CR49] Tsikas D, Hanff E, Bollenbach A, Kruger R, Pham VV, Chobanyan-Jürgens K, Wedekind D, Arndt T, Jörns A, Berbée JFP, Princen HMG, Lücke T, Mariotti F, Huneau JF, Ückert S, Frölich JC, Lenzen S (2018). Results, meta-analysis and a first evaluation of UNOxR, the urinary nitrate-to-nitrite molar ratio, as a measure of nitrite reabsorption in experimental and clinical settings. Amino Acids.

[CR50] Vreugdenhil G, Swaak AJ (1990). Effects of oral N-acetylcysteine on gold levels in rheumatoid arthritis. Br J Rheumatol.

[CR51] Wyman B, Perl A (2020). Metabolic pathways mediate pathogenesis and offer targets for treatment in rheumatic diseases. Curr Opin Rheumatol.

[CR52] Yeh YT, Liang CC, Chang CL, Hsu CY, Li PC (2020). Increased risk of knee osteoarthritis in patients using oral N-acetylcysteine: a nationwide cohort study. BMC Musculoskelet Disord.

[CR53] Yi D, Hou Y, Wang L, Long M, Hu S, Mei H, Yan L, Hu CA, Wu G (2016). *N*-Acetylcysteine stimulates protein synthesis in enterocytes independently of glutathione synthesis. Amino Acids.

[CR54] Zhang Q, Ju Y, Ma Y, Wang T (2018). N-acetylcysteine improves oxidative stress and inflammatory response in patients with community acquired pneumonia: a randomized controlled trial. Medicine (baltimore).

